# Supplementary material for: PTEN regulates starburst amacrine cell dendrite morphology during development
Source: Development. 2026 May 7;153(9):dev204980. doi: 10.1242/dev.204980 (PMC13200731; doi:10.1242/dev.204980)
Supplement: Supplementary information [file develop-153-204980-s1.pdf]

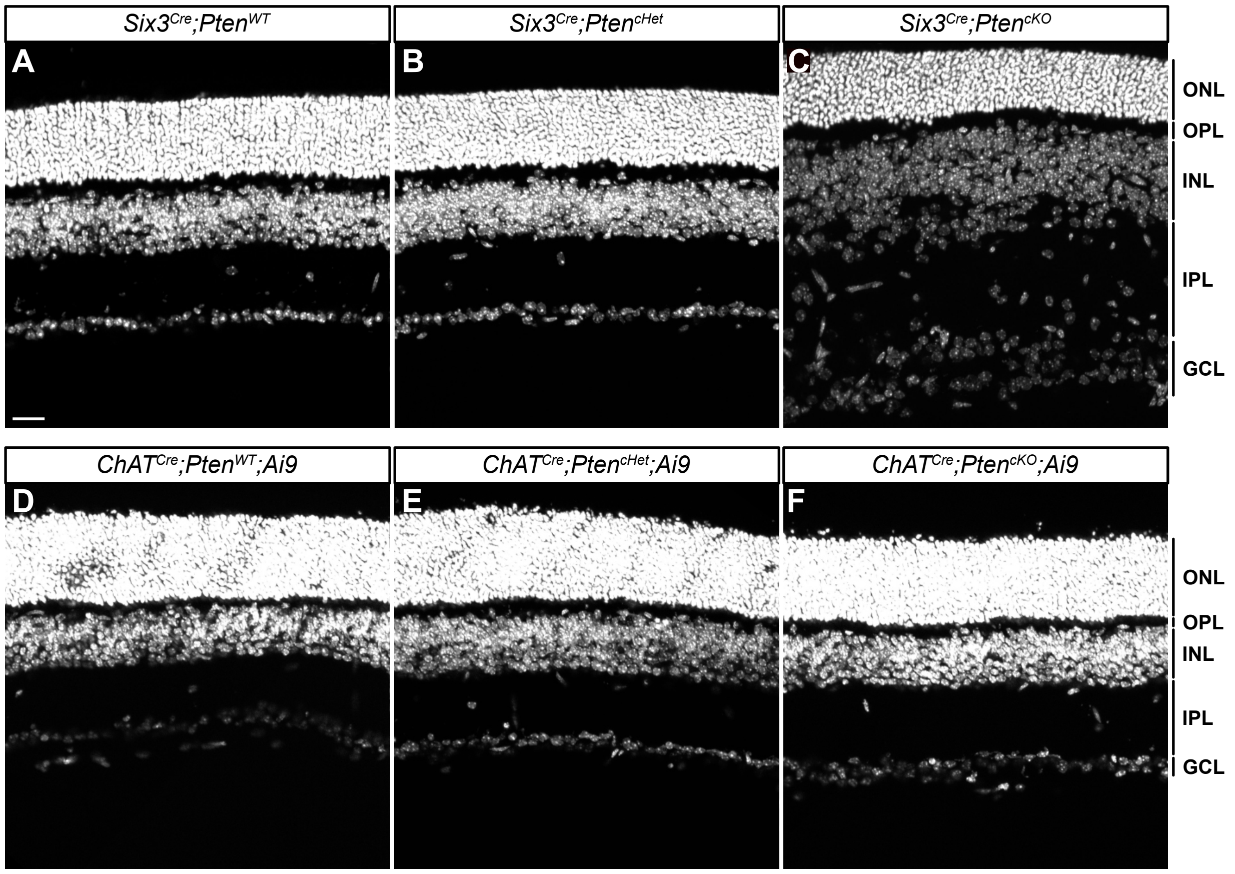

Fig. S1. *ChAT^{Cre};Pten^{cKO}* retinas have grossly normal lamination

A-C. Hoechst staining of P28 *Six^{Cre};Pten^{WT}* (A), *Six^{Cre};Pten^{cHet}* (B), *Six^{Cre};Pten^{cKO}* (C) retinal sections reveal broad disruptions in retinal architecture in *Six^{Cre};Pten^{cKO}* animals. D-F. Hoechst staining of P28 *ChAT^{Cre};Pten^{WT}* (D), *ChAT^{Cre};Pten^{cHet}* (E), *ChAT^{Cre};Pten^{cKO}* (F) retinal sections shows normal lamination in all conditions. SAC-specific deletion of *Pten* does not cause changes to the gross architecture of the retina. Scalebars = 25 µm.

Development • Supplementary information

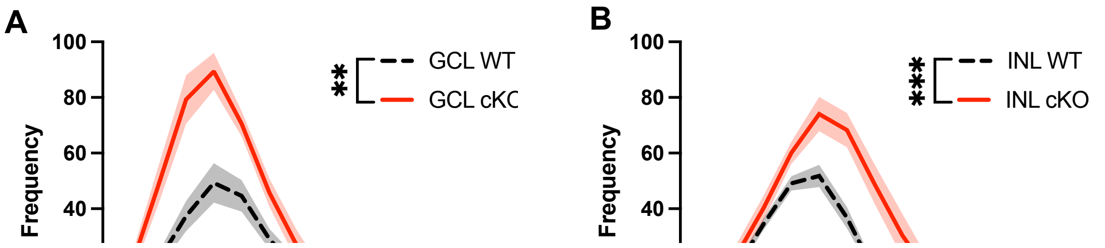

Fig. S2. Branch level analysis in *ChAT*^{Cre}*;Pten*^{cKO} SACs reveal structural differences between GCL and INL SACs

A-B. The distribution of branch levels in individual SACs were tabulated into a histogram for GCL (A) and INL (B) SACs. *ChAT*^{Cre}*;Pten*^{cKO} SACs differ significantly from wildtype controls in both the GCL (*WT: n = 8, cKO: n = 8*) ($p < 0.0001$) and INL (*WT: n = 8, cKO: n = 8*) ($p < 0.0001$). GCL SACs appear to have an enrichment of lower order dendrites, while INL SACs show an enrichment of higher order dendrites. Data reported as mean ± SEM.

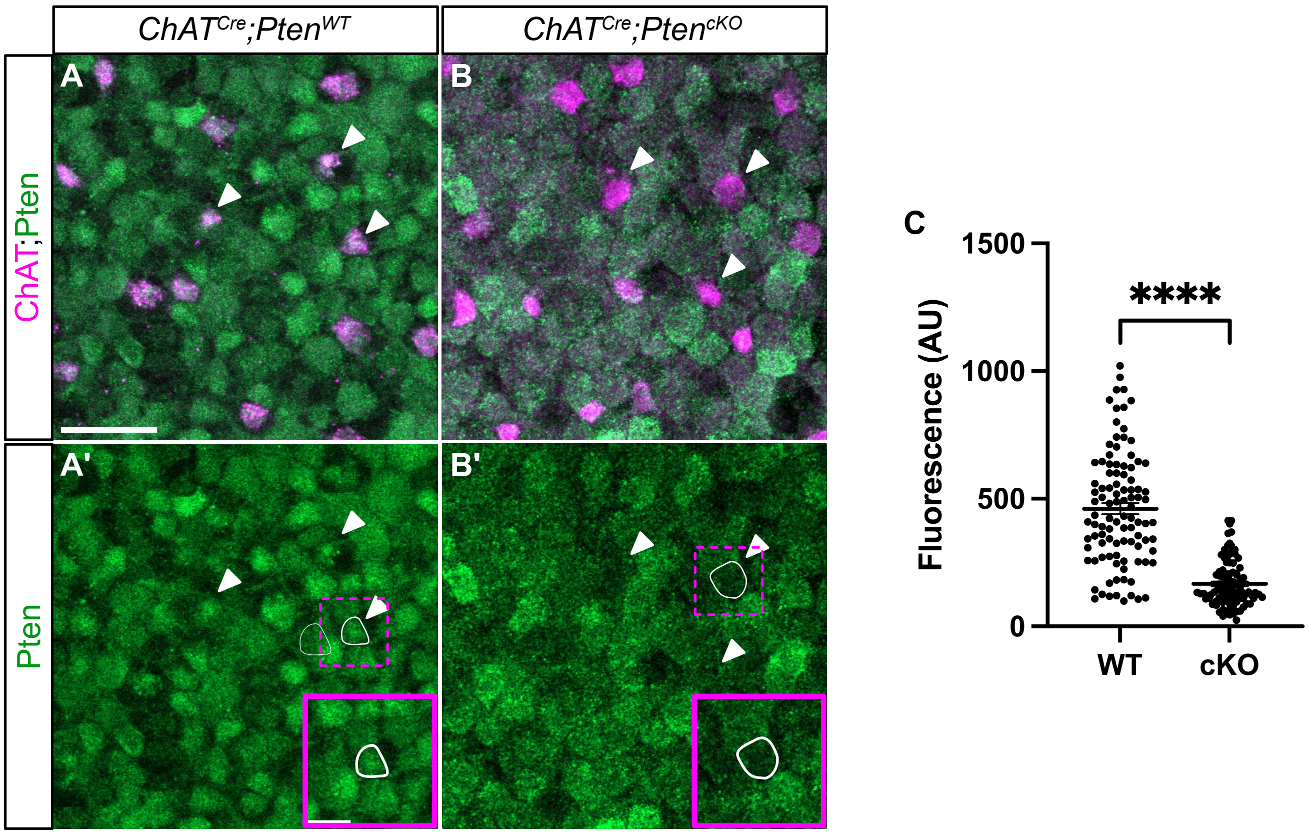

Fig. S3. PTEN levels are significantly reduced by P7 in *ChAT^{Cre};Pten^{cKO}* SACs

A-B'. P7 retinal flatmounts from *ChAT^{Cre};Pten^{WT}* (A) and *ChAT^{Cre};Pten^{cKO}* (B) animals showing ChAT staining of the SAC population and PTEN staining (A'-B'). Insets (pink square) highlight PTEN staining within an individual SAC (white circle). **C.** Quantification of PTEN staining within individual SAC somas. *ChAT^{Cre};Pten^{cKO}* SACs show a significant reduction in PTEN signal compared to controls (*WT: n = 100 cells from 3 animals, 461.0 ± 22.15; cKO: n = 91 cells from 2 animals, 166.4 ± 9.556*) ($p < 0.0001$). Data reported as mean ± SEM. Scalebars in full images = 25 µm. Scalebars in insets = 10 µm.

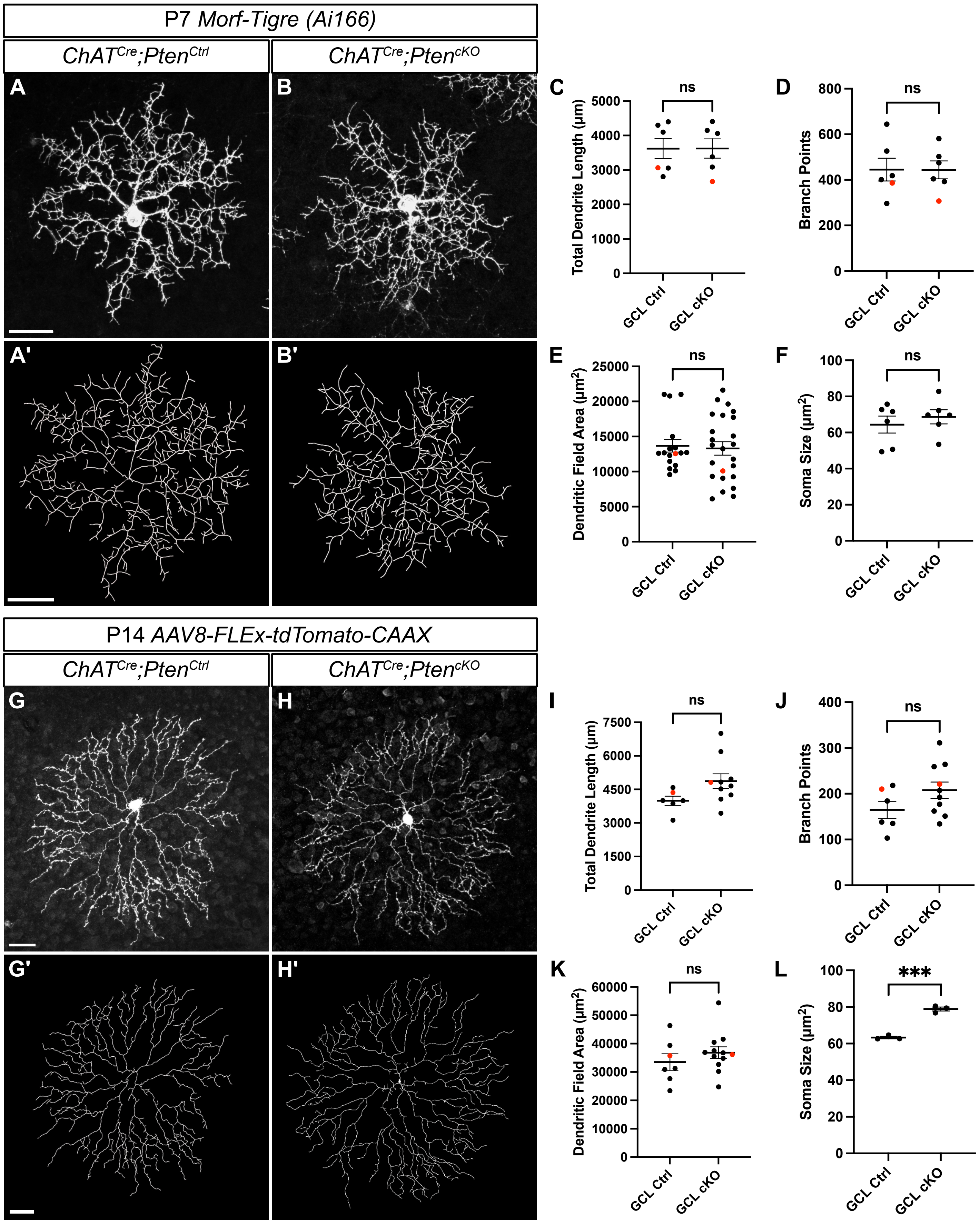
P7 Morf-Tigre (Ai166)
ChATCre;PtenCtrl
ChATCre;PtencKO
A
B
A'
B'
C
Total Dendrite Length (µm)
ns
GCL Ctrl
GCL cKO
D
Branch Points
ns
E
Dendritic Field Area (µm2)
ns
F
Soma Size (µm2)
ns
P14 AAV8-FLEx-tdTomato-CAAX
ChATCre;PtenCtrl
ChATCre;PtencKO
G
H
G'
H'
I
Total Dendrite Length (µm)
ns
J
Branch Points
ns
K
Dendritic Field Area (µm2)
ns
L
Soma Size (µm2)
***

Fig. S4. *Pten* deletion from SACs does not affect their morphology at early developmental timepoints

A-B. P7 SACs from *ChAT*^{Cre}*;Pten*^{cHet}*;Ai166* and *ChAT*^{Cre}*;Pten*^{cKO}*;Ai166* retinas were sparsely labeled using a genetically encoded *Morf-Tigre* reporter. Images show single SACs located in the GCL. **A'-B'.** Imaris reconstructions of P7 SACs from A-B. **C-E.** Quantification of total dendrite length (*Ctrl: n = 6, 3620 ± 293.6; cKO: n = 6, 3623 ± 279.7*) (p = 0.778), number of branch points (*Ctrl: n = 6, 445.2 ± 49.84; cKO: n = 6, 443.5 ± 39.23*) (p = 0.889), and dendritic field area (*Ctrl: n = 17, 13672 ± 900.3; cKO: n = 24, 13297 ± 956.0*) (p = 0.784) from individual SACs showed no significant differences between control and cKO SACs at P7. **F.** Quantification of average soma size (*Ctrl: n = 6, 64.39 ± 4.710; cKO: n = 6, 68.73 ± 3.920*) (p = 0.495) by animal showed no changes between control and cKO SACs. **G-H.** P14 *ChAT*^{Cre}*;Pten*^{cHet} and *ChAT*^{Cre}*;Pten*^{cKO} SACs were sparsely labeled by injection of *AAV8-FLEx-tdTomato-CAAX*. Images show single SACs located in the GCL. **G'-H'.** Imaris reconstructions of P14 SACs from G-H. **I-K.** Quantification of total dendritic length (*Ctrl: n = 6, 3989 ± 205.5; cKO: n = 10, 4873 ± 326.4*) (p = 0.072), number of branch points (*Ctrl: n = 6, 164.7 ± 18.86; cKO: n = 10, 207.8 ± 17.82*) (p = 0.137), dendritic field area (*Ctrl: n = 7, 33546 ± 2898; cKO: n = 12, 36816 ± 2052*) (p = 0.361) from individual SACs showed no significant differences between control and cKO SACs. **L.** Quantification of average soma size revealed significant increases in cKO SACs at P14 (*Ctrl: n = 3, 63.32 ± 0.6511; cKO: n = 3, 78.88 ± 1.092*) (p = 0.0003). Red dots indicate data from representative images. Data reported as mean ± SEM and contain cells from at least 3 animals. Scalebars = 25 µm.

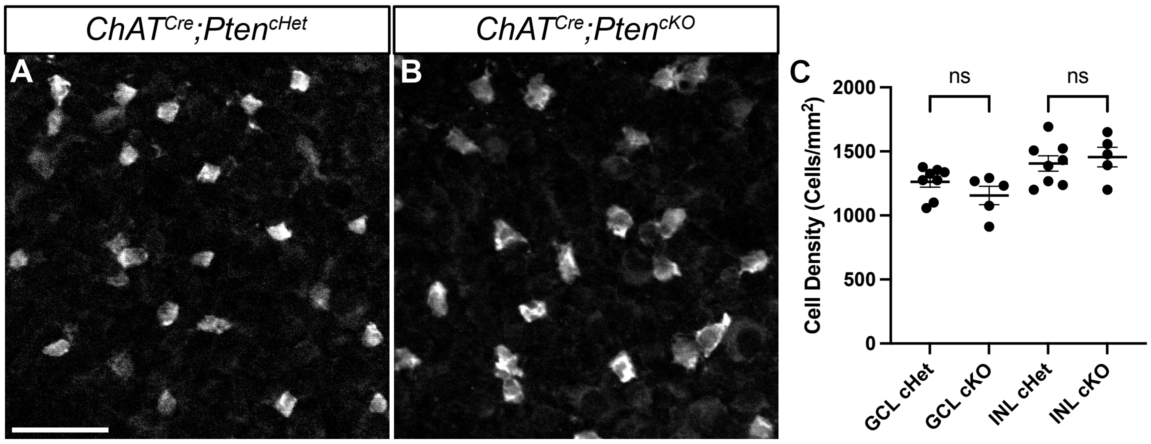

Fig. S5. *ChAT^{Cre}* mediated *Pten* deletion from SACs does not cause changes in cell density at P60

A, B. Representative images of P60 SACs from the GCL labeled with ChAT antibody. **C.** Quantification of cell density reveals no changes in cell density between *ChAT^{Cre};Pten^{cHet}* and *ChAT^{Cre};Pten^{cKO}* SACs at P60 in both the GCL (*cHet: n = 8, 1263 ± 42.23; cKO: n = 5, 1156 ± 71.58*) (p = 0.1931) and INL (*cHet: n = 8, 1406 ± 59.30; cKO: n = 5, 1456 ± 76.89*) (p = 0.6171). Data reported as mean ± SEM. Scalebars = 25 µm.

Development • Supplementary information

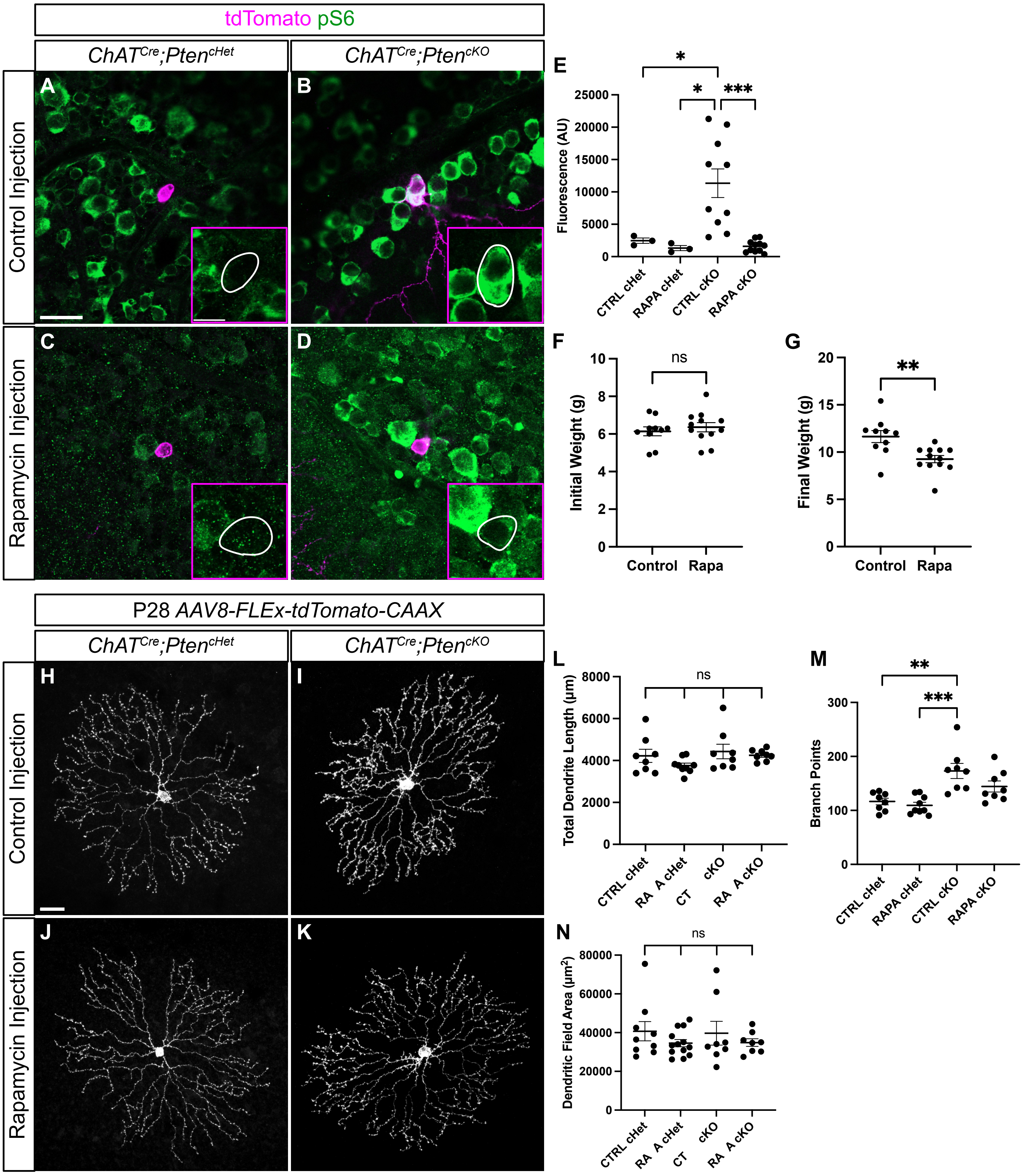

Development • Supplementary information

Fig. S6. Effect of daily rapamycin administration on hypertrophic branching in *ChAT^{Cre};Pten^{cKO}* SACs

A-D. P28 retinal flatmount images from a single z-plane showing a single virally labeled tdTomato+ SAC (magenta) and pS6 immunostaining (green). *ChAT^{Cre};Pten^{cHet}* SACs show no appreciable levels of pS6 in both control (A) and vehicle (C) injection conditions. By contrast, *ChAT^{Cre};Pten^{cKO}* SACs that were given control injections (B) had high levels of pS6, while *ChAT^{Cre};Pten^{cKO}* SACs administered with rapamycin showed a significantly reduced pS6 levels. Insets (pink square) highlight pS6 staining in SACs (white circle). **E.** Quantification of pS6 levels in individual SACs show a significant reduction in rapamycin injected *ChAT^{Cre};Pten^{cKO}* SACs compared with genotype matched control injected SACs (*CTRL cHet: n = 3 cells from 3 animals, 2467 ± 414; RAPA cHet: n = 3 cells from 2 animals, 1330 ± 397.4; CTRL cKO: n = 10 cells from 4 animals, 11347 ± 2210; RAPA cKO: n = 11 cells from 3 animals, 1593 ± 275.1*) (p = 0.0002). *ChAT^{Cre};Pten^{cKO}* SACs did not differ significantly from *ChAT^{Cre};Pten^{cHet}* injected with control (p = 0.990) or rapamycin solutions (p = 0.9997). **F-G.** Quantification of pup weight at first day of injection (F) and final day of injection (G). While there is no difference initially (*Control: n = 10, 6.24 ± 0.2353; Rapa: n = 12, 6.358 ± 0.2432*) (p = 0.5310), after 14 days of injection, mice injected with rapamycin weighed less than controls (*Control: n = 10, 11.64 ± 0.6395; Rapa: n = 12, 9.258 ± 0.3905*) (p = 0.0036). Both sexes were included and no difference was seen between males and females at either timepoint. **H-K.** P28 retinal flatmounts of individually labeled SACs from *ChAT^{Cre};Pten^{cHet}* and *ChAT^{Cre};Pten^{cKO}* animals injected with either control or rapamycin solution. **L-N.** Quantification of morphometric statistics obtained in Imaris show no significant differences in total dendrite length (*CTRL cHet: n = 8 cells from 3 animals, 4223 ± 311.8; RAPA cHet: n = 9 cells from 5 animals, 3756 ± 120.4; CTRL cKO: n = 8 cells from 4 animals, 4430 ± 347.6; RAPA cKO: n = 8 cells from 3 animals, 4253 ± 94.14*) (p = 0.2325) and dendrite field area (*CTRL cHet: n = 8 cells from 3 animals, 40727 ± 4955; RAPA cHet: n = 9 cells from 5 animals, 34556 ± 1865; CTRL*

Development • Supplementary information

cKO: n = 8 cells from 4 animals, 39688 ± 6127; RAPA cKO: n = 8 cells from 3 animals, 34848 ± 1968) (p = 0.5435). There was a significant difference in dendrite branching between the groups (*CTRL cHet: n = 8 cells from 3 animals, 116 ± 6.003; RAPA cHet: n = 9 cells from 5 animals, 109.4 ± 5.701; CTRL cKO: n = 8 cells from 4 animals, 173.1 ± 13.90; RAPA cKO: n = 8 cells from 3 animals, 144.4 ± 10.21*) (p = 0.001). A post-hoc Tukey HSD test revealed differences between control injected $ChAT^{Cre};Pten^{cKO}$ SACs and $ChAT^{Cre};Pten^{cHet}$ SACs independent of injection (*CTRL cHet vs CTRL cKO*: p = 0.0012; *RAPA cHet vs CTRL cKO*: p = 0.0002). Notably, rapamycin injected $ChAT^{Cre}; Pten^{cKO}$ SACs did not show a significant difference between both $ChAT^{Cre};Pten^{cHet}$ SAC groups (*CTRL cHet vs RAPA cKO*: p = 0.1866; *RAPA cHet vs RAPA cKO*: p = 0.0564) and the control injected $ChAT^{Cre};Pten^{cKO}$ SACs (*CTRL cKO vs RAPA cKO*: p = 0.1659). These results show administration of rapamycin attenuates the $ChAT^{Cre};Pten^{cKO}$ hypertrophic branching phenotype. Data reported as mean ± SEM. Scalebars in full images = 25 µm. Scalebars in insets = 10 µm.

Development • Supplementary information

Table S1. Key resources

REAGENT or RESOURCE	SOURCE	IDENTIFIER
Antibodies		
Goat anti-ChAT (1:500, IHC)	Millipore	Cat #: AB144P<br>RRID: AB_11214092
Goat anti-tdTomato (1:1000, IHC)	Biorbyt	Cat #: orb182397<br>RRID: AB_2687917
Rabbit anti-pS6 Ser235/236 (1:100, IHC)	Cell Signaling	Cat #: 81736<br>RRID: AB_2938546
Rabbit anti-pS6 Ser240/244 (1:800, IHC)	Cell Signaling	Cat #: 35708<br>RRID: AB_2938547
Rabbit anti-Pten (1:500, IHC)	Cell Signaling	Cat #: 9559S<br>RRID: AB_10695541
Rabbit anti-GFP (1:1000, IHC)	Abcam	Cat #: ab6556<br>RRID: AB_305564
Chicken anti-GFP (1:500, IHC)	Abcam	Cat #:ab13970<br>RRID:AB_300798
anti-Goat Alexa 546 (1:500, IHC)	Thermo Fisher Scientific	Cat #: A-11056<br>RRID: AB_142628
anti-Rabbit Alexa 488 (1:500, IHC)	Thermo Fisher Scientific	Cat #: A-21206<br>RRID: AB_2535792
anti-Rabbit Alexa 647 (1:500, IHC)	Thermo Fisher Scientific	Cat #: A-31573<br>RRID: AB_2536183
anti-Chicken Alexa 488 (1:500, IHC)	Thermo Fisher Scientific	Cat #: A78948<br>RRID: AB_2921070
Bacterial and virus strains		
AAV8-FLEx-tdTomato-CAAX		
AAV1-FLEx-mGFP-2A-Synaptophysin-mRuby (Beier et al. 2015)	Addgene	Plasmid #: 71760-AAV1<br>RRID: Addgene_71760
Chemicals, peptides, and recombinant proteins		
16% Paraformaldehyde	Fisher	Cat #: 50-980-487
Glyoxal	Fisher	Cat #: AC156225000
Sodium Azide	Fisher	Cat #: S227I-100
Triton X-100	Fisher	Cat #: BP151-100
Agarose	Fisher	Cat #: BP1356-500
Ethidium Bromide 1% Solution	Fisher	Cat #: BP1302-10
Normal Donkey Serum	Fisher	Cat #: 017-000-121
DreamTaq	Fisher	Cat #: FERK1082
Fluoromount-G	Fisher	Cat #: OB100-01
Neg-50	Fisher	Cat #: 22-046-511
2-methylbutane	Fisher	Cat #: AA19387AY
Sucrose	Fisher	Cat #: BP220-1
Acetic Acid, Glacial	Fisher	Cat #: A35-500
Hoechst 33342 (1:5000 IHC)	Fisher	Cat #: H3570
Ames' Medium	Sigma	Cat #: A1420-10X1L
Rapamycin	Fisher	Cat #: AAJ62473MC
Polythylene glycol 400	Fisher	Cat #: B21992.30
Tween 80	Fisher	Cat #: T0546500G
Bacteriostatic 0.9% Sodium Chloride	Hospira	Cat #: 00409196607
Experimental models: Organisms/strains		
Mus Musculus: **C57BL/6J**	The Jackson Laboratory	Strain #: 000664<br>RRID: IMSR_JAX:000664

Development • Supplementary information

ChAT^{Cre} (Rossi et al. 2011)<br>**B6.129S-*Chat$^{tm1(cre)Lowl}$*/MwarJ**	The Jackson Laboratory	Strain #: 031661<br>RRID: IMSR_JAX:031661
Six3^{Cre} (Furuta et al. 2000)<br>**Tg(Six3-cre)69Frty/GcoJ**	The Jackson Laboratory	Strain #: 019755<br>RRID: IMSR_JAX:019755
Pten^{flox} (Backman et al. 2001)<br>**B6.129S4-*Pten^{tm1Hwu}*/J**	The Jackson Laboratory	Strain #: 006440<br>RRID: IMSR_JAX:006440
Ai9 (Madisen et al. 2010)<br>**B6.Cg-*Gt(ROSA)26Sor$^{tm9(CAG\text{-}tdTomato)Hze}$*/J**	The Jackson Laboratory	Strain #: 007909<br>RRID: IMSR_JAX:007909
TIGRE-MORF (Veldman et al. 2020)<br>**B6;129S-*Igs7$^{tm166(tetO\text{-}EGFP*,CAG\text{-}tTA2)Hze}$*/XwyJ**	The Jackson Laboratory	Strain #: 035404<br>RRID: IMSR_JAX:035404
TCF/Lef:H2B/GFP (Ferrer-Vaquer et al. 2010)<br>**Tg(TCF/Lef1-HIST1H2BB/EGFP)61Hadj/J**	The Jackson Laboratory	Strain #: 013752<br>RRID: IMSR_JAX:013752
Oligonucleotides		
Genotyping primers: Cre Forward 5'-tgccacgaccaagtgacagcaatg-3' and Cre Reverse 5'-accagagacggaaatccatcgctc-3'	Integrated DNA Technologies	N/A
Genotyping primers: Pten Flox Forward 5'-CAAGCACTCTGCGAACTGAG-3' and Pten Flox Reverse 5'-AAGTTTTTGAAGGCAAGATGC-3'	Integrated DNA Technologies	N/A
Genotyping primers: GFP Forward 5'-CTACGGCGTGCAGTGCTTC-3' and GFP Reverse 5'-CTGGGTGCTCAGGTAGTG-3'	Integrated DNA Technologies	N/A
Genotyping primers: MORF3 WT Forward 5'-CTGGCTTCTGAGGACCG-3' and MORF3 WT Reverse 5'-AATCTGTGGGAAGTCTTGTCC-3'	Integrated DNA Technologies	https://www.jax.org/Protocol?stockNumber=035403&protocolID=39963
Genotyping primers: MORF3 MUT Forward 5'-ACCACTATCAGCAGAATACGC-3' and MORF3 MUT Reverse 5'-AATTCGGCCATGTTGTTGTC-3'	Integrated DNA Technologies	https://www.jax.org/Protocol?stockNumber=035403&protocolID=39963
Software and algorithms		
Fiji (ImageJ)	Schindelin et al. 2012	https://imagej.net/software/fiji/<br>RRID: SCR_002285
Graphpad Prism 9	Graphpad Software	https://www.graphpad.com/<br>RRID: SCR_002798
Imaris 10.2.0	Bitplane	https://imaris.oxinst.com/<br>RRID: SCR_007370
IPLaminator	Li et al. 2016	https://isoptera.lcsc.edu/IPLaminator/
WinDRP	Rodieck 1991	N/A
MATLAB	MathWorks	RRID:SCR_001622
ZEN Blue	Zeiss	RRID: SCR_013672

Development • Supplementary information
